# Attitude and Behaviors towards SARS-CoV-2 Vaccination among Healthcare Workers: A Cross-Sectional Study from Poland

**DOI:** 10.3390/vaccines9030218

**Published:** 2021-03-04

**Authors:** Bartosz Szmyd, Filip Franciszek Karuga, Adrian Bartoszek, Katarzyna Staniecka, Natalia Siwecka, Agnieszka Bartoszek, Maciej Błaszczyk, Maciej Radek

**Affiliations:** 1 Department of Neurosurgery, Spine and Peripheral Nerves Surgery, Medical University of Lodz, 90-549 Łódź, Poland; bartoszmyd@gmail.com (B.S.); adrian.bartoszek@stud.umed.lodz.pl (A.B.); katarzyna.staniecka@stud.umed.lodz.pl (K.S.); natalia.siwecka@stud.umed.lodz.pl (N.S.); maciej.blaszczyk@umed.lodz.pl (M.B.); maciej.radek@umed.lodz.pl (M.R.); 2 Department of Sleep Medicine and Metabolic Disorders, Medical University of Lodz, 92-215 Łódz, Poland; 3 Department of Pathophysiology, Medical University of Lublin, 20-090 Lublin, Poland; 4 Department of Clinical Chemistry and Biochemistry, Medical University of Lodz, 90-419 Łódź, Poland; 5 Department of Family Medicine and Community Nursing, Medical University of Lublin, 20-081 Lublin, Poland; agabartoszek@wp.pl

**Keywords:** COVID-19, SARS-CoV-2, vaccination, healthcare workers, depression, anxiety, stress

## Abstract

Healthcare workers are particularly exposed to biological risk during their daily occupational activities. Nowadays, severe acute respiratory syndrome coronavirus 2 (SARS-CoV-2) has become one of the most widespread infectious agents. In the current study, we performed a survey on the attitude and behavior of Polish healthcare workers (HCW), which comprise physicians (MD) and administrative healthcare assistants (HA) towards the Coronavirus Disease 2019 (COVID-19) vaccination. Our study involved 2300 subjects (42.17% female; 10.96% MD; 5.87% HA). The evaluation was conducted using a Google Forms survey based on original questions and the Depression, Anxiety and Stress Scale—21 Items questionnaire. HCW significantly more often demonstrated their willingness to get vaccinated against the SARS-CoV-2 as compared to the control group (82.95% vs. 54.31%, respectively). The main concern, as regards all groups, was the development of long-term side effects after getting COVID-19 vaccine. The study revealed that depression significantly affects the willingness to get vaccinated. The readiness was significantly strengthened by positive medical history of recommended vaccinations, fear of catching COVID-19, as well as fear of passing on the disease to the relatives. Overall, the percentage of HCW, who want to be vaccinated against COVID-19 remains unsatisfactory. Further works exploring this subject are needed to take a step closer to achieving the herd immunity in the era of the COVID-19 pandemic.

## 1. Introduction

The several cases of severe pneumonia of unknown origin that were observed in Wuhan at the end of 2019 have initiated one of the most important events of this century. The condition was referred to as Coronavirus Disease 2019 (COVID-19), and it was established that the underlying cause of the disease constitutes a novel coronavirus, classified as severe acute respiratory syndrome coronavirus 2 (SARS-CoV-2) [1]. Exponentially increasing incidence of COVID-19, as observed both in China and worldwide, led to the announcement on COVID-19 pandemic by the World Health Organization in March 2020 [1]. Thus, a race against time in the middle of death and devastation has begun [2]. As it was put by Khuroo et al., “no drug has the power to fight the infection and bring normalcy to the utter chaos”. The only solution for the remaining problem seems to be an effective and safe vaccine, available at an affordable price [2].

Finally, in December 2020, the Food and Drug Administration issued the first Emergency Use Authorization for COVID-19 vaccine in individuals 16 years of age and older [3]. As healthcare workers (HCW) and medical students are particularly exposed to biological risk during their everyday occupations, vaccination strategies developed by proper government institutions worldwide have indicated them as the first group to get the vaccine. Previously published papers revealed that the coverage for recommended vaccination among healthcare workers is generally <30%, despite confirmed effectiveness and safety of the vaccines [4]. Thus, we would like to assess which factors affect the attitude and behaviors towards SARS-CoV-2 vaccination among healthcare workers.

Our previous survey study performed on medical students has proven that the willingness to get vaccinated against SARS-CoV-2 as soon as possible depends on several factors: fear of passing on the disease to relatives, fear of long-term side effects on a scale of 0-10, and the presence of the depression symptoms in the past week [5]. In the current study we hypothesize that: (1) healthcare workers present pro-vaccination behaviors and attitudes significantly more often than the control group, (2) depression, anxiety and stress level affect the willingness to be vaccinated in study participants.

## 2. Materials and Methods

### 2.1. Study Design and Participants

We have performed an online survey study among Polish healthcare workers during the public debate on SARC-CoV-2 vaccination (see Supplementary Materials). Self-administered online questionnaire created in Google Forms was available from 22 December 2020 to 8 January 2021. The respondents were divided into 2 main groups: HCW and control (CG). HCW is an umbrella term which relates to people of various healthcare-related professions [4]. In our study, HCW group comprised physicians (MD) and administrative healthcare assistants (HA). HCW participants were invited to participate in the study through a link to the survey, that was delivered via:

- the researchers’ University and/or hospitals’ emails and official websites and social media profiles (Facebook) of the following institutions: Medical University of Lodz, Central Teaching Hospital of the Medical University of Lodz, as well as University Hospital WAM-CSW
- social media profile of “Porozumienie Rezydentów”—one of the biggest organizations that gathers Polish HCWs

At the same time, CG were recruited using a link to the survey, that was available on the social media of the science channel called “emce kwadrat”.

### 2.2. Measurement Tools

We performed the survey based on a literature review and Depression, Anxiety and Stress Scale (DASS-21), which is 21 items questionnaire. The general part included questions about age, gender, financial income, professional status, former SARS-CoV-2 infection among participants and their relatives, fear of being infected with SARS-CoV-2, population size of the place of residence and work, and the former experience with vaccination among participants and their relatives. The second part, addressed to HCW, was focused on the participants’ grades in microbiology, clinical immunology, pediatrics, and infectious diseases courses.

The last, third part comprised the standardized DASS-21 survey, that is widely used in the Polish population. The scale was used to measure the intensification of depression, anxiety and stress among study participants. Each item of that tool is rated on a scale of 0–3, and the total score ranges from 0 to 63, with the higher scores indicating more severe depression, anxiety and/or stress symptoms. The internal consistency of DASS-21, assessed with Cronbach’s α, was found to be sufficient for the study (Cronbach’s α = 0.928).

### 2.3. Data Collection

All participants have given an informed consent to participate in the study. Confidentiality and anonymity were maintained, as no data that could help to identify a responder were collected. The local Bioethics Committee confirmed that, according to Polish law and Good Clinical Practice regulations, this research does not require an approval of a Bioethics Committee (KB nr 542/20) [6].

### 2.4. Sample Size Calculation and Statistical Analysis

We have decided to perform the study for18 days or more, so as to obtain the required minimum sample size. Thus, the preventive sample size calculation was performed using a calculator provided by The University of British Columbia [7], which is based on the formula available at ‘Fundamentals of Biostatistics’ by Rosner [8].

(1) $$n_{1}\;=\;{\left(\frac{\sqrt{\bar{p}*\bar{q}*\left(1+\frac{1}{k}\right)}*z_{1-\frac{\alpha }{2}}\;+\;\sqrt{p_{1}*q_{1}+\frac{p_{1}*q_{1}}{k}*z_{1-\beta }}}{p_{2}-p_{1}}\right)}^{2}$$

(2) $$n_{1}\;=\;k*n_{1}$$

where: *α*—significance level, 1—*β*—power, *k*—*n*_2_ is *k*—times as large as *n*_1_, *p*_1_, *p*_2_—projected true probabilities of success in the two groups.

(3) $$q_{1},q_{2}\;=\;1-p_{1},1-p_{2};$$

(4) $$\bar{p}\;=\;\frac{p_{1}+k*p_{2}}{1+k},\;\bar{q}\;=\;1-\;\bar{p}$$

The percentage of medical doctors and control group participants who wanted to be vaccinated was calculated based on the first fifty questionnaires, separately for each group (96% and 58%, respectively). For standard *k* = 1, α = 0.05, and power of 0.80, the minimal size of each group was 19.

The data collected were verified for completeness, quality, and consistency, as in our previous study [6]. Statistical analysis was performed using STATISTICA 13.1 (TIBCO, Palo Alto, Santa Clara, CA, USA). A level of 5% was used as a significance threshold for all the results, unless stated otherwise. In the case of multiple testing, the Bonferroni correction was used. No data obtained had normal distribution (Shapiro–Wilk’s test, *p* > 0.05), and thus, they were reported as a median (1. quartile—3. quartile) [9]. The relationship between the independent subgroups were assessed using Mann–Whitney U test. Multiple testing of continuous variables was performed using analysis of variance (ANOVA)/Kruskal–Wallis test with proper post hoc testing. The qualitative data, as presented by n (%), were analyzed using chi-square test, chi-square test with Yates’ correction, or Fisher’s test based on the size of the smallest subgroup (*n* ≥ 15, 15 > *n* ≥ 5, 5 > *n*, respectively) [10]. The binary logistic regression was performed to assess the eventual facilitators/barriers, that could affect the willingness to be vaccinated among HCW [10]. The regression model was built based on the univariate analysis and further adjusted to the baseline characteristics of the enrolled subjects.

## 3. Results

### 3.1. Study Group

The study comprised 2300 respondents: 1913 (83.17%) CG, 10.96% MD, and 5.87% HA. The median age was 26.94 and 31.39, respectively. Most of them studied in the cities with over 500,000 residents: 159 (63.10%)—MD, 51 (37.78%)—HA, and 712 (37.22%). The details are presented in Table 1.

### 3.2. Experiences with COVID-19 and the Related Anxiety

The results obtained showed that MD were tested for SARS-CoV-2 infection significantly more often than CG and HA (74.60% vs. 21.85%, *p* < 0.001 and 74.60% vs. 31.85%, *p* < 0.001, respectively). Simultaneously, MD had higher frequency of positive results comparing to both CG and HA (21.43% vs. 8.31% and 21.43% vs. 6.67%, respectively). Despite the percentage of family members with confirmed SARS-CoV-2 infection among MD was higher than in CG and HA groups (67.46% vs. 54.73 and 67.46% vs. 51.11%, respectively), the rate of COVID-19-related deaths was not statistically significant between groups. Generally, MD were more concerned about both contracting SARS-Cov-2 infection and infecting their elderly relatives, comparing to CG and HA. In all groups, the main COVID-19-related concerns were health deterioration in family members, post-COVID syndrome, and deterioration of their own health. More detailed information about experiences with COVID-19 and the related anxiety among CG and HCW are shown in Table 2.

### 3.3. Vaccination-Related Experiences and Anxiety

MD participating in the study significantly more often declared a desire to get vaccinated against the SARS-CoV-2 with the messenger ribonucleic acid (mRNA)-based vaccines, comparing to both CG and HA (94.44% vs. 54.31% and 94.44% vs. 61.48%, respectively) [11]. By contrast, both CG and HA were more worried about vaccination side effects in comparison to MD, despite that the MD group and their relatives had experienced vaccine-related side effects in the past more often (25% vs. 13.96% and 27.38% vs. 18.82%, respectively). The main concern regarding vaccination in all groups proved to be the long-term side effects of the vaccine. HCW used both mandatory and recommended vaccination more frequently than CG (98.41% vs. 88.08% and 77.38% vs. 31.26%, respectively). More detailed information about experiences and anxiety related to COVID-19 vaccination are presented in Table 3.

### 3.4. Mental Well-Being According to the DASS-21 Questionnaire

According to the DASS-21 questionnaire, HW obtained higher scores in the area of depression comparing to CG: 6 (3–13) vs. 4 (2–9), *p* < 0.001. No differences were observed in the terms of anxiety and stress between the investigated groups (Table 4).

Moreover, we have prepared logistic regression model to assess the common impact of these psychological parameters on the willingness to get vaccinated. This approach revealed that the willingness is significantly affected either by depression (OR = 0.973, 95% CI: 0.953–0.992, *p* = 0.008) or stress level (OR = 1.049, 95% CI: 1.022–1.075, *p* < 0.001) adjusted to other psychological parameters. See Table 5 for further information.

### 3.5. Factors Influencing Pro-Vaccination Attitudes

The logistic regression revealed that the willingness to get vaccinated is significantly strengthened by the positive history of recommended vaccinations (OR = 2.082, 95% CI: 1.453–2.982, *p* < 0.001), the fear of COVID-19 (OR = 1.560, 95% CI: 1.429–1.701, *p* < 0.001), of passing on the disease to relatives (OR = 1.306, 95% CI: 1.219–1.398, *p* < 0.001), and the depression symptoms in the past week (OR = 1.050, 95% CI: 1.011–1.089, *p* = 0.011). When the fear of vaccination side-effects grows, the readiness for vaccination is reduced (OR = 0.564, 95% CI: 0.531–0.598, *p* < 0.001). See Table 6. for further information.

Moreover, in MD group, the grades obtained in the courses where vaccinology played a key role were not significantly different in those MD who wanted to get vaccinated and those who did not. Seniority also did not differ in MD in terms of the willingness to get vaccinated (*p* = 0.537). What is more, among CG, higher earnings were associated with the desire to get vaccinated (3623 (2700–5500) vs. 3300 (2500–5000); *p* < 0.001). Considering HCW, both MD and HA, higher income was not related to the willingness to get vaccinated.

## 4. Discussion

It is a well-known fact that physicians play a crucial role during the COVID-19 pandemic. However, without the support of people of the other healthcare occupations, their work would be significantly hindered. In particular, the administrative staff of hospitals, ambulatory and medical universities belong to the mentioned group. Possibly, the lack of appropriate medical education as well as increased exposition of this group to infectious material makes them one of the possible infectious vectors, including SARS-CoV-2 [4,12,13]. Moreover, the level of vaccination coverage among HCW for other infectious diseases like flu, rubella, tetanus, etc. was also found not to be satisfactory [4,14]. To the best of our knowledge, this is one of the first studies assessing MD and HA attitudes towards COVID-19 vaccination in the era of public debate on safety and necessity of getting the vaccine [15].

### 4.1. Attitude to the Risk Associated with SARS-CoV-2 Pandemic

Since the pandemic has started, more than 2 million of deaths from SARS-CoV-2 and multiple complications (for example post-COVID-19 syndrome) have been registered [16]. Anxiety among people may stem from the fear of infection, but also from the other concerns such as isolation, job loss, problems with education and social stigma [6,15,17].

Medical professions constitute an exceptional occupation in the time of the pandemic. Due to a higher risk of direct COVID-19 exposure, they are at higher risk of infection [18]. Additionally, MD more often face higher stress levels, due to multiple duties, possible isolation from family, and traumatic experiences in the medical practice. This may result in health decline, depression, and problems in private life [19]. A common consequence of that is a possibility of developing professional burnout [20]. Moreover, families of physicians are also more exposed to COVID-19-related inconveniences; indeed, in our study, physician’s families suffered more often from SARS-CoV-2 comparing to CG and HA, even though physicians visited their close ones less frequently.

Non-clinical healthcare workers, for example administrative workers in hospitals, are also in the group at higher risk of COVID-19 infection due to their daily contact with medical personnel and patients [13,19]. However, in our study, the increased rate of infections in HA was not detected, even though the group was tested more frequently than CG. One of the reasons for that phenomenon might be the facilitated access to good-quality preventive measures (e.g., masks with filters, disinfectants) and the adequate knowledge of their usage [21].

Due to increased risk of infection and any related inconveniences, HCW tend to fear the infection significantly more often than the control group. Relatively low fear of infection in the control group may arise from lack of knowledge about the course and complications of the disease and from the belief in conspiracy theories; the latter postulate that the government and medical groups exaggerate the pandemic, or that the pandemic in fact does not exist [22,23]. The most common concern in all groups was fear of worsening of health in family members, which was substantially higher among physicians and administrative workers in comparison to the control group. Significant difference was also observed in the level of fear of infecting relatives in people who had already suffered from SARS-CoV-2 infection. Among MD, this fear was significantly lower than in CG. It might be caused by the better knowledge about antibody formation mechanism and its significance in the process of gaining immunity for viruses between HCW [24].

Unfortunately, due to the possibility of being potential vectors for the infection [25], healthcare workers experienced stigma across the world, including Poland. Mentioned situations were especially common during the first phase of the pandemic. Despite that, the physicians who took part in our study constituted the group that was the least concerned with social stigma problem.

### 4.2. Willingness to Get Vaccinated

More than 94% of physicians were willing to be vaccinated against SARS-CoV-2 (88.5% as soon as possible); this is in accordance with the official statistical data on the utilization of COVID-19 vaccines amongst Polish healthcare workers, that was announced by the Polish Ministry of Health on the 1 February 2021 [26]. Administrative workers declared their intention to vaccinate at the level of 61.5%, whilst in the control group—54%, about half of which indicated for getting vaccine at an undefined “later” time. These results are consistent with the work of Lazarus et al., who examined willingness to get vaccinated in June 2020 in the polish population [27]. At that time, only about 56% of the respondents were willing to receive a potential vaccination, giving Poland the penultimate place on the list of 19 surveyed countries. In addition, in the same survey, 182 out of 666 Poles refused to get vaccination, which was the highest proportion of negative responses among 19 countries [27]. On the other hand, the results of the survey conducted by Feleszko et al. on over 1000 Poles were less consistent with our findings. Only 37% of respondents declared willingness to vaccinate, 34% were undecided and 28% did not want to be vaccinated. However, the worst results were noted in Turkey and the Czech Republic, where more than 40% of the respondents were against COVID-19 vaccination. These results are very alarming, as they may threaten the inability to develop herd immunity, which is a prerequisite for controlling the epidemic of coronavirus [28].

Higher willingness to vaccinate among doctors and medical students fits well with the vaccination schedule, as they belong to the group of the highest priority for vaccination in Poland [5,29]. That gives time for media campaigns that aim to convince the undecided groups until it is to be their turn. Not only does the vaccination of medical staff and students gain the context of reducing the spread of infections (epidemics), but it also sets an example for uncertain citizens who need to have the efficacy and safety of vaccines confirmed [30]. Although the administrative staff also belong to the priority group in Poland, their attitude raises concern, as up to 38.5% of them refuse to vaccinate. In the previous study on the influenza vaccines by Dini et al., participants pursuing the occupation of a nurse were significantly less eager to vaccinate against influenza [4]. In view of the above, the mentioned HCW subgroups may constitute potential in-hospital vectors that can generate problems in controlling the epidemics in health-care facilities [12,13]. Thus, the importance of their decisions should not be marginalized.

One of the key elements to achieve herd immunity is to better understand the concerns of the undecided part of the population and then to convince them to get vaccinated V.

### 4.3. Concerns about COVID-19 Vaccination and Conspiracy Theories

The level of fear related to the vaccine side effects was the lowest among MD, but it was gradually increasing in the control group, to reach the peak among the administrative staff. Long-term complications proved to be the main concern in all the investigated groups. In the study by Di Martino et al., in which the data were collected from August to November 2019, only 1.6% of doctors were wary of the long-term health effects of vaccinations, whilst in our study, the percentage of doctors who were afraid of long-term side effects of COVID-19 vaccination was 32.14% [14]. Significant differences between the doctors’ group and the control group could be seen in their respective beliefs in conspiracy theories and autism-inducing effect of the vaccine [31]. Among the doctors, such concerns did not exist, or they were significantly less frequent as compared with the control group. Unfortunately, the belief in conspiracy theories in HA was similar to that observed in the CG. Due to the fact, that those people might not follow implemented personal safety measures, thinking that they are excessive and unnecessary, they may cause a serious threat to the society in the epidemic. The other phenomena such as misinformation about vaccines and conspiracy theories that are widely spread among people are also very harmful to the society [32]. The examples include claiming that the virus was a Chinese engineered bioweapon, or that there is an association between high-band 5G frequencies and COVID-19 incidence [33,34]. The assessment of the actual scale of this problem was also addressed in the study by Daniel Freeman et al., which has demonstrated that around 50% of the surveyed English citizens exhibited some belief in conspiracy theories, while around 25% showed a full support for such theories [35].

Aversion to vaccines is primarily caused by spreading false information, mainly via social media platforms and with the help of anti-vaccination groups [32,36]. The biggest fears include vaccination side effects and doubts about the effectiveness of vaccination [30]. One widely spread theory claims that herd immunity does not exist, whilst the other that the vaccines cause autism or that the pandemic is in fact created by the media [30,37]. As they become more and more prevalent, depending on personal interpretation and some emotional aspects, they seem to be more compelling to the majority of the society, as compared to the raw and objective scientific facts [38,39]. The lack of support for such claims in the MD group may be attributed to their medical knowledge and expertise in vaccinology.

### 4.4. Factors Influencing Positive Attitudes towards Vaccination

According to the study by Lazarus et al., in the countries with high levels of social trust in government (e.g., China, South Korea, Singapore), the approval rate of the COVID-19 vaccine exceeded 80% [27]. This finding indicates that increase in reliability of government might have a beneficial influence on the attitude towards COVID-19 vaccination within the society. However, the recommendations and adequate explanation provided by the respected HCWs may also play an important role [4].

In the study by Feleszko et al., 301 respondents who had refused to be vaccinated were additionally asked what could change their mind, and more than half of them claimed that under no circumstances would their change their mind [28]. However, 17% of them could have been convinced with the presentation of the results of scientific research on vaccine safety, 11% would vaccinate themselves to enter foreign countries which would require that, and 10% would take a vaccine if there would be a high fine (1500 €) for not vaccinating themselves or their children. It has also been shown that, with increasing age, the task to convince or encourage vaccine-sceptics to get vaccinated gets more difficult [28].

In the binary logistic regression model applied in our study, we have detected a variety of significant parameters that increase the willingness to get vaccinated among study participants; they include fear of COVID-19, fear of passing the disease to relatives, and past medical history of recommended vaccination (e.g., for influenza). The results of the study performed by Dini et al. were consistent with our findings, as they revealed that the history of influenza vaccinations was associated with an increased adherence to vaccination [4]. Surprisingly, depression also positively influenced the willingness to get vaccinated, while stress was found to be insignificant. Fear of vaccination side effect was the strongest factor that negatively influenced the willingness to vaccinate, thus the social campaigns explaining the nature and incidence of vaccine related side-effect could substantially reduce the skepticism about COVID-19 vaccination [40].

### 4.5. Comparison between the Present and Previous Study on Medical and Non-Medical Students

In our previous study that was performed on medical and non-medical students, we applied the analogous questionnaire, which was available from 22 December 2020 to 25 December 2020 [5]. Of all the groups included in the two studies, medical students and doctors were the most comparable ones. Even so, doctors showed more willingness to be vaccinated against COVID-19 (94.44% vs. 91.99%), less fear of vaccine side effects, as well as they less frequently believed in conspiracy theories (3.17% vs. 8.69%) than medical students. None of the doctors admitted believing in the injection of microchips with the vaccine or autism associated with vaccination. On the other hand, in the case of medical students, 1.75% of them believed in microchip injection and 3.93% in autism associated with vaccination. Moreover, it was observed that with the increasing year of medical studies, the willingness to get vaccinated also increased These observations provide support to the hypothesis that the clinical experience is effective at forming a pro-vaccination attitude. Paradoxically, despite the fact that medical students were less involved in the fight against COVID-19 pandemic, they turned out to be more depressed and stressed compared to doctors [41].

The control groups (non-medical students and non-healthcare workers) of the two studies were also relatively similar to each other. However, students turned out to be more likely to get vaccinated than workers (59.42% vs. 54.31%), in spite of a similar severity of fear of vaccination side effects in both groups. The possible reason for this phenomenon could be a lesser tendency to believe in conspiracy theories in the students group (19.55% vs. 21.48%). In addition, students tended to be more depressed and anxious, while workers were significantly more stressed.

## 5. Conclusions

Fear of COVID-19 vaccine side-effects and belief in conspiracy theories are a real threat to the public safety in achieving herd immunity. Medical administrative workers turned out to be vulnerable to these threats, in contrast to physicians. This observation is disturbing in the context of spread of the disease in the health facilities, and it should not be overlooked. To reduce the phenomenon of hesitancy to vaccinate, it may be reasonable to, firstly, understand its causes and, secondly, implement appropriately designed social campaign. Fear of long-term complications and other possible COVID-19 side effects is the strongest negative factor influencing willingness to get vaccinated. As it is a modifiable factor, the actions to reassure society about the safety of COVID-19 vaccination could be of special benefit.

## 6. Strength and Limitations

This is one of the very first studies, performed either in Poland or worldwide, that explores the attitudes and behaviors towards SARS-CoV-2 vaccination among physicians and medical administrators. The data were obtained during a heated public debate on SARS-CoV-2 vaccination. The median of depression, anxiety and stress level were assessed in large group of participants. Additionally, a calculation of the group size was performed.

We have observed the following limitations in our study: Firstly, it was an online survey, accessible through a link. Thus, only people with Internet access could take part in our study. Moreover, we have no information about the response rate. Secondly, the group of participants who want to receive the vaccination might have been over-represented in the questionnaire study. Thirdly, this was only a nationwide study, and hence the obtained results cannot be related to the other groups/nations. Finally, we have not taken into consideration the impact of commonly used drugs on the participants’ mental well-being [42].

## Figures and Tables

**Table 1:** Study and control group characteristics (*n* = 2300).

	Healthcare Workers			Control Group	*p*-Value
	Doctors	Administrative	Total		
Total; *n*	252 (10.96%)	135 (5.87%)	387	1913 (83.17%)	Not applicable
Male; *n* (%)	71 (28.17%)	51 (37.78%)	122 (31.52%)	1208 (63.15%)	*** p * < 0.001 ** *, *, *, *
Mean age	29.87 (SD = 6.99)	34.26 (SD = 9.97)	31.39 (SD = 8.42)	26.94 (SD = 9.05)	*** p * < 0.001 ** *, *, -, *

In bold—statistically significant differences; “*”—statistically significant differences in post hoc assessment; “-“—lack of this differences; post hoc results are presented as follows: Doctors vs. Control group, Administrative vs. Control group, Doctors vs. Administrative group, Total vs. Control group.

**Table 2:** Experiences with COVID-19 and the related anxiety among control group and healthcare workers.

	Healthcare Workers *n* (%)			Control Group *n* (%)	*p*-Value
	Doctors	Administrative	Total		
Previous SARS-CoV-2 infection	54 (21.43%)	9 (6.67%)	63 (16.28%)	159 (8.31%)	<0.001 *, -, *, *
Tested for SARS-CoV-2	188 (74.60%)	43 (31.85%)	231 (59.69%)	418 (21.85%)	<0.001 *, *, *, *
Tested for SARS-CoV-2 ^: PCR:					
Nose	89 (35.32%)	17 (12.59%)	106 (27.39%)	126 (6.59%)	<0.001 *, *, *, *
Mouth	34 (13.49%)	6 (4.44%)	40 (10.34%)	79 (4.13%)	<0.001 *, -, *, *
Mouth and nose	77 (30.56%)	16 (11.85%)	93 (24.03%)	150 (7.84%)	<0.001 *, -, *, *
Quick antigen test	40 (15.87%)	7 (5.19%)	47 (12.14%)	51 (2.67%)	<0.001 *, -, *, *
ELISA	35 (13.89%)	10 (7.41%)	45 (11.63%)	60 (3.14%)	<0.001 *, *, -, *
Family member with confirmed SARS-CoV-2 infection	170 (67.46%)	69 (51.11%)	239 (61.76%)	1047 (54.73%)	<0.001 *, -, *, -
Family member deceased in the course of COVID-19	16 (6.35%)	4 (2.96%)	20 (5.17%)	122 (6.38%)	0.362
How often do you visit elderly family members:					
Never	56 (22.22%)	25 (18.52%)	81 (20.93%)	354 (18.5%)	0.413
<1×/month	102 (40.48%)	46 (34.07%)	148 (38.24%)	653 (34.13%)	0.130
1–2×/month	52 (20.63%)	19 (14.07%)	71 (18.35%)	369 (19.29%)	0.428
3–10×/month	30 (11.9%)	27 (20.00%)	57 (14.73%)	303 (15.84%)	0.178
>10×/month	10 (3.97%)	17 (12.59%)	27 (6.98%)	213 (11.13%)	<0.001 *, -, *, *
Fear of contracting SARS-CoV-2 on a 10-point scale:					
General	6 (3–7)	5 (3–7)	5 (3–7)	4 (2–6)	<0.001 *, *, -, *
After illness	5 (3–7)	4 (2–6)	5 (3–6.25)	3 (2–5)	0.151
Main COVID-19-related concerns ^					
Health or academic problems	62 (24.60%)	24 (17.78%)	86 (22.22%)	329 (17.20%)	0.008 *, -, -, *
Health deterioration	143 (56.75%)	71 (52.59%)	214 (55.30%)	842 (44.01%)	<0.001 *, -, -, *
Post-COVID syndrome	124 (49.21%)	62 (45.93%)	186 (48.06%)	855 (44.69%)	0.407
Health deterioration in family members	184 (73.02%)	88 (65.19%)	272 (70.28%)	1069 (55.88%)	<**0.001** *, *, -, *
Social stigma	5 (1.98%)	7 (5.19%)	12 (3.10%)	159 (8.31%)	<0.001 *, *, -, *
How concerned are you about passing on the disease to your relatives on a scale of 0–10?					
Overall	8 (7–9)	7 (4–8)	8 (6–9)	6 (3–8)	<0.001 *, -, *, *
After recovering from COVID	2 (0–3)	2 (2–4)	2 (0.5–3)	4 (1–7)	<0.001 *, -, -, *

In bold—statistically significant differences; “*”—statistically significant differences in post hoc assessment; “-“—lack of this differences; post hoc results are presented as follows: Doctors vs. Control group, Administrative vs. Control group, Doctors vs. Administrative group, Total vs. Control group; ^—multiple-choice.

**Table 3:** Experiences and anxiety related to vaccination among control group and healthcare workers.

	Healthcare Workers			Control Group	*p*-Value
	Doctors	Administrative	Total		
Do you plan to get vaccinated?					
Yes—overall	238 (94.44%)	83 (61.48%)	321 (82.95%)	1039 (54.31%)	<0.001 *, -, *, *
As soon as possible	223 (88.49%)	62 (45.93%)	285 (73.64%)	561 (29.33%)	<0.001 *, *, *, *
At some point in the future	15 (5.95%)	21 (15.56%)	36 (9.30%)	478 (24.99%)	<0.001 *, *, *, *
No	14 (5.56%)	52 (38.52%)	66 (17.05%)	873 (45.64%)	<0.001 *, -, *, *
I do not know	3 (1.19%)	20 (14.81)	23 (5.94%)	380 (19.86%)	<0.001 *, -, *, *
How much are you worried about vaccination side effects on a scale of 0–10?					
Overall	2 (1–3)	5 (2–8)	2 (1–5)	4 (1–8)	<0.001 *, -, *, *
After previous SARS-CoV-2 infection	2 (0–3)	2 (2–4)	2 (0.5–3)	4 (1–7)	<0.001 *, *, -, *
What concerns you the most about getting vaccination? ^					
Severe hypersensitivity reaction	26 (10.32%)	10 (7.41%)	36 (9.30%)	141 (7.37%)	0.280
Fever and malaise	34 (13.49%)	24 (17.78%)	58 (14.99%)	247 (12.91%)	0.332
Swelling and reddening around point of injection	13 (5.16%)	6 (4.44%)	19 (4.91%)	106 (5.54%)	0.914
Long-term complications	81 (32.14%)	60 (44.44%)	141 (36.43%)	847 (44.28%)	<0.001 *, -, *, *
Conspiracy theories (overall):	8 (3.17%)	22 (16.3%)	30 (7.75%)	411 (21.48%)	<0.001 *, -, *, *
Microchip injection	0 (0%)	5 (3.7%)	5 (1.29%)	127 (6.64%)	<0.001 *, -, *, *
Belief that herd immunity does not exist	2 (0.79%)	3 (2.22%)	5 (1.29%)	59 (3.08%)	0.051
Limitation of civil rights	6 (2.38%)	18 (13.33%)	24 (6.2%)	299 (15.63%)	<0.001 *, -, *, *
Control of births by vaccine manufacturers	3 (1.19%)	9 (6.67%)	12 (3.10%)	124 (6.48%)	<0.001 *, -, *, *
Autism	0 (0%)	8 (5.93%)	8 (2.07%)	80 (4.18%)	0.003 *, -, *, -
Have you ever experienced any vaccination side effects?	63 (25%)	20 (14.81%)	83 (21.45%)	267 (13.96%)	<0.001 *, -, -, *
If so, which one of the following ^:					
Local reaction	53 (21.03%)	13 (6.63%)	66 (17.05%)	131 (6.85%)	<0.001 *, -, -, *
Fever, malaise	40 (15.87%)	12 (8.89%)	52 (13.44%)	171 (8.94%)	<0.001 *, -, -, *
Severe reaction	0 (0%)	2 (1.48%)	2 (0.52%)	16 (0.84%)	0.623
Long-term side effects	0 (0%)	3 (2.22%)	3 (0.78%)	30 (1.57%)	0.256
Has anyone from your family experienced any side effects of vaccines?	69 (27.38%)	28 (20.74%)	97 (25.06%)	360 (18.82%)	0.001 *, -, -, *
If so, which one of the following ^:					
Local reaction	52 (20.63%)	14 (10.37%)	66 (17.05%)	137 (7.16%)	<0.001 *, -, *, *
Fever, malaise	51 (20.24%)	18 (13.33%)	69 (17.83%)	219 (11.45%)	<0.001 *, -, -, *
Severe reaction	4 (1.53%)	6 (4.44%)	10 (2.58%)	56 (2.93%)	0.416
Long-term side effects	1 (0.40%)	5 (3.7%)	6 (1.55%)	57 (2.98%)	0.040 *, -, *, -
Past medical history of mandatory vaccinations:					**Complete vs. rest** <0.001 *, -, *, *
Complete	248 (98.41%)	116 (85.93%)	364 (96.06%)	1685 (88.08%)	
Incomplete	4 (1.59%)	14 (10.37%)	18 (4.65%)	201 (10.51%)	
None	0 (0%)	5 (3.7%)	5 (1.29%)	27 (1.41%)	
Past medical history of recommended vaccinations, *n* (%)	195 (77.38%)	40 (29.63%)	235 (60.72%)	598 (31.26%)	<0.001 *, -, *, *

In bold—statistically significant differences; “*”—statistically significant differences in post hoc assessment; “-“—lack of this differences; post hoc results are presented as follows: Doctors vs. Control group, Administrative vs. Control group, Doctors vs. Administrative group, Total vs. Control group. ^—multiple-choice.

**Table 4:** Mental well-being according to the DASS-21 questionnaire among control group and healthcare workers.

	Healthcare Workers			Control Group	*p*-Value
	Doctors	Administrative	Total		
Depression	5 (2–9)	5 (2–9)	6 (3–13)	4 (2–9)	<0.001 *, *, -, *
Anxiety	3 (1–6)	3 (1–6)	3 (1–6)	2 (1–5)	0.633
Stress	6 (3.5–10.5)	6 (4–11)	6 (3–11)	7 (4–12)	0.640

In bold—statistically significant differences; “*”—statistically significant differences in post hoc assessment; “-“—lack of this differences; post hoc results are presented as follows: Doctors vs. Control group, Administrative vs. Control group, Doctors vs. Administrative group, Total vs. Control group.

**Table 5:** The binary logistic regression model assessing the impact of DASS-21 parameters on the willingness to get vaccinated (total: healthcare workers and control group).

	OR	95%CI	*p*-Value
Intercept	0.537	0.463–0.622	** <0.001 **
Depression	0.973	0.953–0.992	** 0.008 **
Anxiety	0.984	0.954–1.013	0.280
Stress	1.049	1.022–1.075	** <0.001 **

In bold—statistically significant differences.

**Table 6:** The logistic regression model evaluated the impact of tested parameters on the willingness to get vaccinated adjusted to the baseline characteristics of the enrolled subjects.

Binary Logistic Regression Model			
	OR	95%CI	*p*-Value
Intercept	1.049	0.527–2.088	0.891
Sex (male)	0.890	0.631–1.255	0.508
Family member with confirmed SARS-CoV-2 infection (Yes)	1.281	0.925–1.771	0.135
Family member deceased in the course of COVID-19 (Yes)	0.807	0.388–1.675	0.566
Past medical history of recommended vaccinations (Yes)	2.082	1.453–2.982	** <0.001 **
Age (years)	1.013	0.997–1.029	0.095
The fear of COVID-19 (0–10)	1.560	1.429–1.701	** <0.001 **
The fear of passing on the disease to relatives (0–10)	1.306	1.219–1.398	** <0.001 **
The fear of vaccination side-effects (0–10)	0.564	0.531–0.598	** <0.001 **
Depression	1.050	1.011–1.089	** 0.011 **
Stress	0.964	0.923–1.006	0.096

In bold—statistically significant differences.

## Data Availability

Data are available upon request (Filip Karuga; filipfranciszek439@gmail.com).

## Supplementary Materials

The following are available online at https://www.mdpi.com/2076-393X/9/3/218/s1, Questionnaire 1: The questionnaire used in the study [the original, Polish version], Questionnaire 2: The questionnaire used in the study [English translation].
