# Supplementary material for: Attitude and Behaviors towards SARS-CoV-2 Vaccination among Healthcare Workers: A Cross-Sectional Study from Poland
Source: Vaccines (Basel). 2021 Mar 4;9(3):218. doi: 10.3390/vaccines9030218 (PMC8000513; doi:10.3390/vaccines9030218)
Supplement: Supplementary file 1 [file vaccines-09-00218-s001.pdf]

Supplementary material 1. The questionnaire used in the study [the original, Polish version]:

Szczepienia przeciwko SARS-CoV-2

Wstęp

Szanowni Państwo, z góry dziękujemy za Państwa wkład w rozwój wiedzy na temat szczepień przeciwko wirusowi SARS-Cov-2. Wiemy jak cenna jest każda z poświęconych nam minut, dlatego postaraliśmy się ograniczyć pytania do tych najważniejszych. Podzieliliśmy je dla Państwa na trzy sekcje. W pierwszej części skupimy się na Państwa podejściu do SARS-CoV-2 i szczepień. Kolejna część pytań będzie zindywidualizowaną serią pytań, ukierunkowaną na szczegóły Państwa zawodu. W ostatniej, trzeciej części, korzystając ze standardowego kwestionariusza DASS-21 ocenimy natężenie depresji, lęku i stresu.
Pytania obowiązkowe zaznaczono za pomocą gwiazdki (*).

CZĘŚĆ I – Pytania podstawowe

W tej części pytania skupiają się na Państwa podejściu do SARS-CoV-2 i szczepień.

P: Ile ma Pan(i) lat? * *(typ: liczba)*
O: ☐ *(Twoja odpowiedź)*

P: Płeć * *(typ: jednokrotny wybór)*
O: jedna z poniższych:
- Kobieta
- Mężczyzna

P: Czy zdiagnozowano u Pan(i) infekcję wirusem SARS-CoV-2? * *(typ: jednokrotny wybór)*
O: jedna z poniższych:
- Tak
- Nie

P: W jaki sposób próbowano wykryć u Pani/Pana wirusa SARS-CoV-2? * *(typ: wielokrotny wybór/tekst)*
O: wiele odpowiedzi możliwych:
- PCR - wymaz pobierany tylko przez nos
- PCR - wymaz pobierany tylko przez usta
- Opcja PCR - wymaz pobierany zarówno przez nos, jak i usta
- Szybkie testy antygenowe (wynik od razu)
- Przeciwciała metodą ELISA (pobranie krwi, wynik po pewnym czasie)
- Nie miałam/miałem wykonywanych testów
- Inne: ☐ *(Twoja odpowiedź)*

P: Czy ktoś z Pani/Pana bliskich był zakażony wirusem SARS-CoV-2? * *(typ: jednokrotny wybór)*
O: jedna z poniższych:
- Tak
- Nie

P: Czy ktoś z Pani/Pana bliskich zmarł z powodu zakażenia wirusem SARS-CoV-2? * *(typ: jednokrotny wybór)*
O: jedna z poniższych:
Tak
Nie

P: W jakiej miejscowości Pan(i) pracuje/studiuje? * *(typ: jednokrotny wybór)*
O: jedna z poniższych:
Miasto powyżej 500 tys osób
Miasto powyżej 250 tys
Miasto powyżej 100 tys
Miasto powyżej 50 tys
Miasto poniżej 50 tys
Wieś

P: W jakiej miejscowości Pan(i) się wychowywał(a)? * *(typ: jednokrotny wybór)*
O: jedna z poniższych:
Miasto powyżej 500 tys osób
Miasto powyżej 250 tys
Miasto powyżej 100 tys
Miasto powyżej 50 tys
Miasto poniżej 50 tys
Wieś

P: Jak często odwiedza Pan(i) osoby starsze ze swojej rodziny? *(typ: jednokrotny wybór)*
O: jedna z poniższych:
Nie odwiedzam
Rzadziej niż raz na miesiąc
1-2x/miesiąc
3-10x/miesiąc
>10x/miesiąc

P: Czy zamierza się Pan(/i) zaszczepić się przeciwko wirusowi SARS-CoV-2? * *(typ: jednokrotny wybór)*
O: jedna z poniższych:
Tak w najbliższym możliwym terminie/już się zaszczepiłem(am)
Tak, ale za pewien czas
Nie wiem
Nie
Nie, wolę przechorować

P: Jak bardzo w skali od 0-10 obawia się Pan(i) zachorowania na COVID? * *(typ: jednokrotny wybór)*
O: jedna z poniższych:
0 – W ogóle się nie boję
1
2

3
4
5
6
7
8
9
10 – Paniczny lęk

P: Czego najbardziej boi się Pan(i) w związku z zachorowaniem na COVID? *(typ: wielokrotny wybór/tekst)*
O: wiele odpowiedzi możliwych:
Problemy zawodowe/akademickie
Pogorszenie mojego stanu zdrowia
Zespół pocovidowy - wieloukładowy zespół zapalny występujący w następstwie zakażenia koronawirusem. Rozwija się dwa do czterech tygodni po zakażeniu wirusem SARS-CoV-2. Dotyka również pacjentów, którzy przeszli zakażenie bezobjawowo.
Pogorszenie stanu zdrowia najbliższych
Odrzucenie społeczne (piętnowanie)
Inne: *(Twoja odpowiedź)*

P: Jak bardzo w skali od 0-10 obawia się Pan(i) bycia przyczyną zachorowania Pan(i) najbliższych (rodziny, współpracowników)? * *(typ: jednokrotny wybór)*
O: jedna z poniższych:
0 – W ogóle się nie boję
1
2
3
4
5
6
7
8
9
10 – Paniczny lęk

P: Jak bardzo w skali od 0-10 obawia się Pan(i) powikłań poszczepiennych? * *(typ: jednokrotny wybór)*
O: jedna z poniższych:
0 – W ogóle się nie boję
1
2
3
4
5
6

7
8
9
10 – Paniczny lęk

P: Z czego wynikają Pani/Pana niechęć/obawy przed szczepieniem? *(typ: wielokrotny wybór/tekst)*
O: wiele odpowiedzi możliwych:
Ciężka nadwrażliwość na szczepionki
Gorączka, złe samopoczucie
Obrzęk, zaczerwienienie w miejscu wkłucia
Długofalowe powikłania
Możliwość wszepienia chipu
Odporność zbiorowa nie istnieje
Ograniczenie praw obywatelskich
Próba kontroli liczby urodzeń przez producentów
Ryzyko autyzmu
Inne: *(Twoja odpowiedź)*

P: Czy do tej pory wystąpił u Pana/Pani niepożądany odczyn poszczepienny? * *(typ: jednokrotny wybór)*
O: jedna z poniższych:
Tak
Nie

P: Jeśli tak, to jaki/jakie? *(typ: wielokrotny wybór/tekst)*
O: wiele odpowiedzi możliwych:
Miejscowy odczyn niepożądany
Gorączka, gorsze samopoczucie
Ciężki odczyn poszczepienny
Długotrwałe efekty niepożądane
Inne: *(Twoja odpowiedź)*

P: Czy do tej pory wystąpił u kogokolwiek z Pani/Pana bliskich niepożądany odczyn poszczepienny? * *(typ: jednokrotny wybór)*
O: jedna z poniższych:
Tak
Nie

P: Jeśli u Pani/Pana najbliższych wystąpił niepożądany odczyn poszczepienny, to proszę zaznaczyć jaki: *(typ: wielokrotny wybór/tekst)*
O: wiele odpowiedzi możliwych:
Miejscowy odczyn niepożądany
Gorączka, gorsze samopoczucie
Ciężki odczyn poszczepienny
Długotrwałe efekty niepożądane

Nie pamiętam
Inne: (Twoja odpowiedź)

P: Czy do tej pory szczepił(a) się Pan(i) szczepieniami obowiązkowymi? * *(typ: jednokrotny wybór)*
O: jedna z poniższych:
Tak, wszystkie
Tak, ale nie wszystkimi
Nie

P: Czy do tej pory szczepił(a) się Pan(i) szczepieniami zalecanymi (np. przeciwko grypie)? * *(typ: jednokrotny wybór)*
O: jedna z poniższych:
Tak
Nie

P: Jeśli posiada Pan(i) dzieci, to czy były szczepione zgodnie z kalendarzem szczepień? * *(typ: jednokrotny wybór)*
O: jedna z poniższych:
Tak
Nie
Nie posiadam potomstwa

P: Czy jest Pan(i) studentem? * *(typ: jednokrotny wybór)*
O: jedna z poniższych:
Tak
Nie

CZĘŚĆ IIA – Studenci – (Strona 1) Kierunek studiów

P: Jaki jest Pani/Pana kierunek studiów? * *(typ: jednokrotny wybór)*
O: jedna z poniższych:
Niemedyczny
Diagnostyka laboratoryjna
Dietetyka
Farmacja
Fizjoterapia
Medycyna
Stomatologia
Pielęgniarstwo
Położnictwo
Ratownictwo medyczne

P: Jakie są Pana/i średnie miesięczne wydatki? *(typ: liczba)*
O: (Twoja odpowiedź)

CZĘŚĆ IIA – Studenci – (Strona 2) Część szczegółowa ***(tylko dla studentów kierunków medycznych)***

P: Na którym jesteś roku? * *(typ: jednokrotny wybór)*
O: jedna z poniższych:
- I
- II
- III
- IV
- V

P: Jaką średnią uzyskał(a) Pan(i) za kurs mikrobiologii? * *(typ: jednokrotny wybór)*
O: jedna z poniższych:
- 2.0
- 3.0
- 3.5
- 4.0
- 4.5
- 5.0
- Jestem w trakcie kursu
- Kurs rozpocznę na kolejnych latach
- Nie pamiętam
- Nie dotyczy

P: Jaką średnią uzyskał(a) Pan(i) za kurs chorób zakaźnych? * *(typ: jednokrotny wybór)*
O: jedna z poniższych:
- 2.0
- 3.0
- 3.5
- 4.0
- 4.5
- 5.0
- Jestem w trakcie kursu
- Kurs rozpocznę na kolejnych latach
- Nie pamiętam
- Nie dotyczy

P: Jaką średnią uzyskał(a) Pan(i) za kurs immunologii? * *(typ: jednokrotny wybór)*
O: jedna z poniższych:
- 2.0
- 3.0
- 3.5
- 4.0
- 4.5
- 5.0
- Jestem w trakcie kursu
- Kurs rozpocznę na kolejnych latach

Nie pamiętam
Nie dotyczy

P: Jaką średnią uzyskał(a) Pan(i) za kurs propedeutyki pediatrii? * *(typ: jednokrotny wybór)*
O: jedna z poniższych:
2.0
3.0
3.5
4.0
4.5
5.0
Jestem w trakcie kursu
Kurs rozpocznę na kolejnych latach
Nie pamiętam
Nie dotyczy

CZĘŚĆ IIB – Pracownicy – (Strona 1) Wykonywany zawód

P: Jaki zawód Pan(i) wykonuje? * *(typ: jednokrotny wybór)*
O: jedna z poniższych:
Zawód niemedyczny
Diagnosta laboratoryjny
Dietetyk
Farmaceuta
Fizjoterapeuta/rehabilitant
Lekarz
Lekarz dentysta
Pielęgniarka
Pracownik administracyjny
Położna
Ratownik Medyczny
Salowa

P: Jaki jest Pani/Pana średni miesięczny dochód netto w złotówkach (na rękę)? Dane proszę podać bez używania spacji, bez podawania jednostki. *(typ: liczba)*
O: *(Twoja odpowiedź)*

CZĘŚĆ IIB – Pracownicy – (Strona 2) Część szczegółowa *(tylko dla pracowników medycznych)*

P: Jak długo Pan(i) pracuje w zawodzie? * *(typ: liczba)*
O: *(Twoja odpowiedź)*

P: W jakiej miejscowości Pan(i) pracuje/studiuje? * *(typ: jednokrotny wybór)*
O: jedna z poniższych:
Miasto powyżej 500 tys osób

Miasto powyżej 250 tys
Miasto powyżej 100 tys
Miasto powyżej 50 tys
Miasto poniżej 50 tys
Wieś

P: Jaki jest obszar pana pracy? * *(tylko dla diagnostów laboratoryjnych/dietetyków)* *(typ: jednokrotny wybór/tekst)*
O: jedna z poniższych:
Analityka ogólna
Badania genetyczne i/lub molekularne
Chemia kliniczna
Hematologia
Mikrobiologia
Serologia
Inne: *(Twoja odpowiedź)*

P: W jakiej jednostce Pan(i) pracuje? * *(tylko dla fizjoterapeutów/rehabilitantów)* *(typ: jednokrotny wybór)*
O: jedna z poniższych:
Szpital
POZ
Prywatny gabinet

P: Gdzie Pan(i) pracuje? * *(tylko dla ratowników medycznych)* *(typ: wielokrotny wybór/tekst)*
O: wiele odpowiedzi możliwych:
Karetka pogotowia
Transport medyczny
SOR
Poza zawodem
Inne: *(Twoja odpowiedź)*

P: Jaką specjalizację Pan(i) posiada lub w trakcie realizacji Pan(i) jest?/Na jakim oddziale Pan(i) pracuje? * *(tylko dla lekarzy/pielęgniarek)* *(typ: wielokrotny wybór/tekst)*
O: wiele odpowiedzi możliwych:
POZ
Alergologia
Anestezjologia i intensywna terapia
Angiologia
Audiologia i foniatria
Balneologia
Chirurgia dziecięca
Torakochirurgia
Chirurgia naczyniowa
Chirurgia onkologiczna
Chirurgia plastyczna

Chirurgia twarzowo-szczękowa
Choroby płuc
Choroby płuc dzieci
Choroby wewnętrzne
Choroby zakaźne
Dermatologia
Diabetologia
Diagnostyka laboratoryjna
Endokrynologia
Epidemiologia
Farmakologia kliniczna
Gastroenterologia
Gastroenterologia dziecięca
Genetyka kliniczna
Geriatria
Ginekologia onkologiczna
Hematologia
Hipertensjologia
Immunologia kliniczna
Intensywna terapia
Kardiochirurgia
Kardiologia
Kardiologia dziecięca
Medycyna lotnicza
Medycyna morska i tropikalna
Medycyna nuklearna
Medycyna paliatywna
Medycyna pracy
Medycyna ratunkowa
Medycyna rodzinna
Medycyna sądowa
Medycyna sportowa
Mikrobiologia lekarska
Nefrologia
Neonatologia
Neurochirurgia
Neurologia
Neurologia dziecięca
Neuropatologia
Okulistyka
Onkologia i hematologia dziecięca
Onkologia
Ortopedia
Otolaryngologia
Otolaryngologia dziecięca
Patomorfologia
Pediatria

Pediatria metaboliczna
Perinatologia
Położnictwo i ginekologia
Psychiatria
Psychiatria dzieci i młodzieży
Radiologia
Radioterapia
Rehabilitacja medyczna
Reumatologia
Seksuologia
Stażysta
Po stażu przed wyborem specjalizacji
Toksykologia kliniczna
Transfuzjologia kliniczna
Urologia
Urologia dziecięca
Zdrowie publiczne
Inne: ______ *(Twoja odpowiedź)*

P: Jakie jest Pana(i) wykształcenie? * *(tylko dla farmaceutów)* *(typ: jednokrotny wybór)*
O: jedna z poniższych:
Magisterium
Doktorat
Habilitacja
Prof. uczelni
Profesor

P: Jakie wykształcenie Pani posiada? * *(tylko dla pielęgniarek/położnych/ratowników medycznych)* *(typ: jednokrotny wybór/tekst)*
O: jedna z poniższych:
Szkoła ponadgimnazjalna
Licencjat
Magisterium
Doktorat
Inne: ______ *(Twoja odpowiedź)*

P: Jakie jest Pan(i) wykształcenie? * *(tylko dla pracowników administracyjnych)* *(typ: jednokrotny wybór/tekst)*
O: jedna z poniższych:
Podstawowe
Gimnazjalne
Ponadgimnazjalne
Wyższe (w trakcie)
Wyższe
Inne: ______ *(Twoja odpowiedź)*

P: Jakie jest Pan(i) wykształcenie? * *(tylko dla salowych)* *(typ: jednokrotny wybór/tekst)*
O: jedna z poniższych:
- Podstawowe
- Gimnazjalne
- Ponadgimnazjalne
- Wyższe (w trakcie)
- Wyższe
- Inne: [ ] *(Twoja odpowiedź)*

P: Jaką średnią uzyskał(a) Pan(i) za studia? (prosimy podawać liczbę z przecinkiem) * *(tylko dla farmaceutów/fizjoterapeutów/rehabilitantów/lekarzy/lekarzy dentystów)* *(typ: liczba)*
O: [ ] *(Twoja odpowiedź)*

P: Jaką średnią uzyskał(a) Pan(i) za kurs mikrobiologii? * *(typ: jednokrotny wybór/tekst)*
O: jedna z poniższych:
- 2.0
- 3.0
- 3.5
- 4.0
- 4.5
- 5.0
- Nie dotyczy
- Nie pamiętam
- Inne: [ ] *(Twoja odpowiedź)*

P: Jaką średnią uzyskał(a) Pan(i) za kurs chorób zakaźnych? * *(typ: jednokrotny wybór/tekst)*
O: jedna z poniższych:
- 2.0
- 3.0
- 3.5
- 4.0
- 4.5
- 5.0
- Nie dotyczy
- Nie pamiętam
- Inne: [ ] *(Twoja odpowiedź)*

P: Jaką średnią uzyskał(a) Pan(i) za kurs immunologii? * *(typ: jednokrotny wybór/tekst)*
O: jedna z poniższych:
- 2.0
- 3.0
- 3.5
- 4.0
- 4.5

5.0
Nie dotyczy
Nie pamiętam
Inne: (Twoja odpowiedź)

P: Jaką średnią uzyskał(a) Pan(i) za kurs propedeutyki pediatrii? * *(typ: jednokrotny wybór/tekst)*
O: jedna z poniższych:
2.0
3.0
3.5
4.0
4.5
5.0
Nie dotyczy
Nie pamiętam
Inne: (Twoja odpowiedź)

CZĘŚĆ III – Jak często w ciągu OSTATNIEGO TYGODNIA odczuwała Pani/odczuwał Pan następujące objawy:

Poniższe pytania pochodzą z kwestionariusza DASS-21. Proszę przeczytać każde zdanie i zaznaczyć odpowiedź, która sugeruje nasilenie danego objawu u Pani/Pana w OSTATNIM TYGODNIU. Nie ma tu prawidłowych lub złych odpowiedzi – są tylko poprawne – Pani/Pana odczucie. Nie poświęcaj zbyt wiele czasu na jakiekolwiek wypowiedzi.

P: Trudno było mi się odprężyć: * *(typ: jednokrotny wybór)*
O: jedna z poniższych:
Nigdy
Czasami
Często
Zawsze/prawie zawsze

P: Odczuwałam/-em suchość w ustach * *(typ: jednokrotny wybór)*
O: jedna z poniższych:
Nigdy
Czasami
Często
Zawsze/prawie zawsze

P: Nie mogłam/-em doświadczyć pozytywnych uczuć * *(typ: jednokrotny wybór)*
O: jedna z poniższych:
Nigdy
Czasami
Często
Zawsze/prawie zawsze

P: Odczuwałam/-em trudność w oddychaniu (np. duszności, przyspieszony oddech bez wcześniejszego wysiłku fizycznego) * *(typ: jednokrotny wybór)*
O: jedna z poniższych:
Nigdy
Czasami
Często
Zawsze/prawie zawsze

P: Trudno było mi znaleźć inicjatywę do działania * *(typ: jednokrotny wybór)*
O: jedna z poniższych:
Nigdy
Czasami
Często
Zawsze/prawie zawsze

P: Miałam/-em skłonności do przesadnego reagowania na różne sytuacje * *(typ: jednokrotny wybór)*
O: jedna z poniższych:
Nigdy
Czasami
Często
Zawsze/prawie zawsze

P: Odczuwałam/-em drżenie np. rąk * *(typ: jednokrotny wybór)*
O: jedna z poniższych:
Nigdy
Czasami
Często
Zawsze/prawie zawsze

P: Czułam/-em, że zużywam dużo nerwowej energii * *(typ: jednokrotny wybór)*
O: jedna z poniższych:
Nigdy
Czasami
Często
Zawsze/prawie zawsze

P: Byłam/-em przestraszony sytuacjami, gdzie mogłem spanikować lub ośmieszyć się * *(typ: jednokrotny wybór)*
O: jedna z poniższych:
Nigdy
Czasami
Często
Zawsze/prawie zawsze

P: Czułam/-em, że nie ma przede mną przyszłości, nic mnie nie czeka w życiu * *(typ: jednokrotny wybór)*

O: jedna z poniższych:
Nigdy
Czasami
Często
Zawsze/prawie zawsze

P: Czułam/-em się pobudzona/-y (niespokojna/-y) * *(typ: jednokrotny wybór)*
O: jedna z poniższych:
Nigdy
Czasami
Często
Zawsze/prawie zawsze

P: Trudno było się zrelaksować * *(typ: jednokrotny wybór)*
O: jedna z poniższych:
Nigdy
Czasami
Często
Zawsze/prawie zawsze

P: Czułam/-em się smutna/-y i przygnębiona/-y * *(typ: jednokrotny wybór)*
O: jedna z poniższych:
Nigdy
Czasami
Często
Zawsze/prawie zawsze

P: Byłam/-em nietolerancyjna/-y do wszystkiego, co uniemożliwiało mi wzięcie udziału w tym, co robiłem * *(typ: jednokrotny wybór)*
O: jedna z poniższych:
Nigdy
Czasami
Często
Zawsze/prawie zawsze

P: Byłam/-em bliski wpadnięciu w panikę * *(typ: jednokrotny wybór)*
O: jedna z poniższych:
Nigdy
Czasami
Często
Zawsze/prawie zawsze

P: Nie byłam/-em zdolny do odczuwania entuzjazmu * *(typ: jednokrotny wybór)*
O: jedna z poniższych:
Nigdy
Czasami
Często

Zawsze/prawie zawsze

P: Czułam/-em, że jestem niewiele warta/-y * *(typ: jednokrotny wybór)*
O: jedna z poniższych:
Nigdy
Czasami
Często
Zawsze/prawie zawsze

P: Czułam/-em, że jestem raczej przewrażliwiona/-y * *(typ: jednokrotny wybór)*
O: jedna z poniższych:
Nigdy
Czasami
Często
Zawsze/prawie zawsze

P: Zdałam/-em sobie sprawę z pracy mojego serca mimo braku wysiłku fizycznego (np. przyspieszone bicie serca, zanik bicia serca) * *(typ: jednokrotny wybór)*
O: jedna z poniższych:
Nigdy
Czasami
Często
Zawsze/prawie zawsze

P: Czułam/-em się przestraszona/-y bez powodu * *(typ: jednokrotny wybór)*
O: jedna z poniższych:
Nigdy
Czasami
Często
Zawsze/prawie zawsze

P: Czułam/-em, że życie nie ma sensu * *(typ: jednokrotny wybór)*
O: jedna z poniższych:
Nigdy
Czasami
Często
Zawsze/prawie zawsze

Supplementary material 2. The questionnaire used in the study [English translation]:

Vaccination against SARS-CoV-2

Introduction

Ladies and gentlemen, we would like to thank you in advance for your contribution to the development of knowledge on vaccination against SARS-CoV-2 infection. We are aware how precious is every minute that you take for our survey, and for this reason we have decided to include herein only the most essential questions. For your convenience, we have divided the survey into three major parts. The first part is focused on your attitude towards SARS-CoV-2 infection and vaccine, whilst the second part is individualized and tailored to the specifics of your occupation. Lastly, in the third part, the levels of depression, anxiety and stress will be assessed by means of the standard DASS-21 questionnaire.
Obligatory questions are indicated with asterisks (*).

PART I – Basic questions

In this part, the questions are focused on your attitude towards SARS-CoV-2 infection and vaccination.

Q: How old are you? * *(type: number)*
A: ☐ *(your answer)*

Q: Sex * *(type: choice)*
A: one of the following:
  Female
  Male

Q: Have you been diagnosed with SARS-CoV-2 infection? * *(type: choice)*
A: one of the following:
  Yes
  No

Q: By which method have you been tested for SARS-CoV-2 infection? * *(type: multiple choice/text)*
A: multiple answers possible:
  PCR – Nasal Swab Test only
  PCR – Oral/Pharyngeal Swab Test only
  PCR – Nasal and Oral/Pharyngeal Swab Test
  Rapid Antigen Test (results available immediately)
  ELISA Antibody Test (blood sample collection, results available after a while)
  I have not been tested for COVID-19
  Others: ☐ *(your answer)*

Q: Has any of your loved ones been infected with SARS-CoV-2? * *(type: choice)*
A: one of the following:
  Yes
  No

Q: Has any of your loved ones died of SARS-CoV-2 infection? * *(type: choice)*
A: one of the following:
  Yes
  No

Q: Where do you work/study? * *(type: choice)*
A: one of the following:
  City of over 500,000 inhabitants
  City of over 250,000 inhabitants
  City of over 100,000 inhabitants
  City of over 50,000 inhabitants
  City of less than 50,000 inhabitants
  Village

Q: Where did you grow up? * *(type: choice)*
A: one of the following:
  City of over 500,000 inhabitants
  City of over 250,000 inhabitants
  City of over 100,000 inhabitants
  City of over 50,000 inhabitants
  City of less than 50,000 inhabitants
  Village

Q: How often do you visit your elderly family members? *(type: choice)*
A: one of the following:
  I do not visit them
  Less than once a month
  1-2 times a month
  3-10 times a month
  More than 10 times a month

Q: Are you going to get vaccinated against SARS-CoV-2? * *(type: choice)*
A: one of the following:
  Yes, as soon as possible/I am already vaccinated
  Yes, but only after some time
  I do not know
  No
  No, I prefer to recover from COVID-19

Q: How much are you afraid of getting COVID-19 on a scale of 0-10? * *(type: choice)*
A: one of the following:
  0 – Not afraid at all
  1
  2
  3
  4
  5

6
7
8
9
10 – Panic fear

Q: What scares you the most about getting COVID-19? *(type: multiple choice/text)*
A: multiple answers possible:
Professional/academic problems
Deterioration of my health
Post-COVID Syndrome – multisystem inflammatory syndrome occurring as a result of SARS-CoV-2 infection, that develops within 2 to 4 weeks after infection, and that also affects the asymptomatic COVID-19 patients
Deterioration of health in my loved ones
Social rejection (social stigma)
Others: ____ *(your answer)*

Q: How much are you afraid of passing COVID-19 to the loved ones (relatives, coworkers) on a scale of 0-10? * *(type: choice)*
A: one of the following:
0 – Not afraid at all
1
2
3
4
5
6
7
8
9
10 – Panic fear

Q: How much are you afraid of side effects of the COVID-19 vaccine on a scale of 0-10? * *(type: choice)*
A: one of the following:
0 – Not afraid at all
1
2
3
4
5
6
7
8
9
10 – Panic fear

Q: What is the reason for your reluctance/concerns regarding COVID-19 vaccination? *(type: multiple choice/text)*
A: multiple answers possible:
Severe allergic reactions to vaccines
Fever, malaise
Swelling or redness at the injection site
Long-term complications
Possibility of chip insertion
Conviction that herd immunity does not exist
Restriction of civil liberties
Attempt of birth control by manufacturers
Risk of developing autism
Others: ______ *(your answer)*

Q: Have you ever experienced side effects of the vaccine? * *(type: choice)*
A: one of the following:
Yes
No

Q: If so, what was it? *(type: multiple choice/text)*
A: multiple answers possible:
Local side effect
Fever, malaise
Severe allergic reaction to vaccine
Long-term complications
Others: ______ *(your answer)*

Q: Has any of your loved ones ever experienced side effects of the vaccine? * *(type: choice)*
A: one of the following:
Yes
No

Q: If any of your loved ones has ever experienced a side effect of the vaccine, please indicate which one that was: *(type: multiple choice/text)*
A: multiple answers possible:
Local side effect
Fever, malaise
Severe allergic reaction to vaccine
Long-term complications
I do not remember
Others: ______ *(your answer)*

Q: Have you already taken the mandatory vaccinations? * *(type: choice)*
A: one of the following:
Yes, all of them

Yes, but not all of them
No

Q: Have you already taken the recommended vaccinations (e.g. against flu)? * *(type: choice)*
A: one of the following:
Yes
No

Q: If you have children, were they given vaccines according to the vaccination schedule? * *(type: choice)*
A: one of the following:
Yes
No
I do not have children

Q: Are you a student? * *(type: choice)*
A: one of the following:
Yes
No

PART IIA – Students – (Page 1) Field of study

Q: What is your field of study? * *(type: choice)*
A: one of the following:
Nonmedical
Laboratory Diagnostics
Dietetics
Pharmacy
Physical Therapy
Medicine
Dentistry
Nursing
Obstetrics
Emergency Medical Services

Q: What are your average monthly expenses? *(type: number)*
A: ______ *(your answer)*

PART IIA – Students – (Page 2) Detail questions *(for medical students only)*

Q: What year are you in? * *(type: choice)*
A: one of the following:
first
second
third
fourth

fifth

Q: What was your grade in microbiology course? * *(type: choice)*
A: one of the following:
2.0
3.0
3.5
4.0
4.5
5.0
I am currently taking the course
I will take the course in the following years
I do not remember
Not applicable

Q: What was your grade in infectious diseases course? * *(type: choice)*
A: one of the following:
2.0
3.0
3.5
4.0
4.5
5.0
I am currently taking the course
I will take the course in the following years
I do not remember
Not applicable

Q: What was your grade in immunology course? * *(type: choice)*
A: one of the following:
2.0
3.0
3.5
4.0
4.5
5.0
I am currently taking the course
I will take the course in the following years
I do not remember
Not applicable

Q: What was your grade in propaedeutics of pediatrics course? * *(type: choice)*
A: one of the following:
2.0
3.0
3.5
4.0

4.5
5.0
I am currently taking the course
I will take the course in the following years
I do not remember
Not applicable

PART IIB – Professionals – (Page 1) The occupation

Q: What is your profession? * *(type: choice)*
A: one of the following:
Non-medical profession
Laboratory diagnostician
Dietitian
Pharmacist
Physiotherapist/rehabilitator
Doctor
Dentist
Nurse
Medical administrative assistants
Midwife
Paramedic
Orderly

Q: What is your average monthly net income in Polish Zloty (PLN)? Please provide the data without spaces or currency units. *(type: number)*
A: *(your answer)*

PART IIB – Professionals – (Page 2) Detail questions ***(for medical workers only)***

Q: How long have you been working in your profession? * *(type: number)*
A: *(your answer)*

Q: Where do you work/study? * *(type: choice)*
A: one of the following:
City of over 500,000 inhabitants
City of over 250,000 inhabitants
City of over 100,000 inhabitants
City of over 50,000 inhabitants
City of less than 50,000 inhabitants
Village

Q: What is your field of work? * *(for laboratory diagnosticians/dietitians only)* *(type: choice/text)*
A: one of the following:
General analytics
Genetic and/or molecular testing

Clinical chemistry
Haematology
Microbiology
Serology
Others: (your answer)

Q: What health facility do you work in? * *(for physiotherapists/rehabilitators only)* *(type: choice)*
A: one of the following:
Hospital
General Medical Services
Private practice

Q: Where do you work? * *(for paramedics only)* *(type: multiple choice/text)*
A: multiple answers possible:
Ambulance
Medical transport
Emergency department
I do not work in my profession
Others: (your answer)

Q: What is the specialty that you are in the course of or that you have completed?/What unit do you work in? * *(for doctors/nurses only)* *(type: multiple choice/text)*
A: multiple answers possible:
General Medical Services
Allergology
Anaesthesiology and intensive care
Angiology
Audiology and phoniatrics
Balneology
Paediatric surgery
Thoracic surgery
Vascular surgery
Surgical Oncology
Surgery
Maxillofacial surgery
Pulmonology
Paediatric pulmonology
Internal medicine
Infectious diseases
Dermatology
Diabetology
Laboratory diagnostics
Endocrinology
Epidemiology
Clinical pharmacology
Gastroenterology

Paediatric gastroenterology
Clinical genetics
Geriatrics
Gynaecologic oncology
Haematology
Hypertensiology
Clinical immunology
Intensive care
Cardiac surgery
Cardiology
Paediatric cardiology
Aviation medicine
Maritime and tropical medicine
Nuclear medicine
Palliative medicine
Occupational medicine
Emergency medicine
Family medicine
Forensic pathology
Sports medicine
Medical microbiology
Nephrology
Neonatology
Neurosurgery
Neurology
Paediatric neurology
Neuropathology
Ophthalmology
Paediatric oncology and haematology
Oncology
Orthopaedics
Otolaryngology
Paediatric otolaryngology
Pathomorphology
Paediatrics
Paediatric metabolic medicine
Perinatology
Obstetrics and gynaecology
Psychiatry
Child and adolescent psychiatry
Radiology
Radiation therapy
Physical medicine and rehabilitation
Rheumatology
Sexology
Medical internship
I am after the internship and before residency

Clinical toxicology
Clinical transfusion medicine
Urology
Paediatric urology
Public health
Others: ______ *(your answer)*

Q: What is your educational qualification? * *(for pharmacists only)* *(type: choice)*
A: one of the following:
Master's degree
PhD
Post-doctoral degree
Associate Professor
Professor

Q: What is your educational qualification? * *(for nurses/midwives/paramedics only)* *(type: choice/text)*
A: one of the following:
High school
Bachelor's degree
Master's degree
PhD
Others: ______ *(your answer)*

Q: What is your educational qualification? * *(for medical administrative assistants/orderlies only)* *(type: choice/text)*
A: one of the following:
Primary education
Lower secondary education
Upper secondary education
Higher education (in progress)
Higher education (completed)
Others: ______ *(your answer)*

Q: What was your grade point average at college? (please provide a number with the decimal point) * *(for pharmacists/physiotherapists/rehabilitators/doctors/dentists only)* *(type: number)*
A: ______ *(your answer)*

Q: What was your grade in microbiology course? * *(type: choice/text)*
A: one of the following:
2.0
3.0
3.5
4.0

4.5
5.0
I do not remember
Not applicable
Others: [ ] *(your answer)*

Q: What was your grade in infectious diseases course? * *(type: choice/text)*
A: one of the following:
2.0
3.0
3.5
4.0
4.5
5.0
I do not remember
Not applicable
Others: [ ] *(your answer)*

Q: What was your grade in immunology course? * *(type: choice/text)*
A: one of the following:
2.0
3.0
3.5
4.0
4.5
5.0
I do not remember
Not applicable
Others: [ ] *(your answer)*

Q: What was your grade in propaedeutics of pediatrics course? * *(type: choice/text)*
A: one of the following:
2.0
3.0
3.5
4.0
4.5
5.0
I do not remember
Not applicable
Others: [ ] *(your answer)*

PART III – How often in the PAST WEEK did you feel the following symptoms:

The following questions are taken from the DASS-21 questionnaire. Please read each statement carefully and tick the answer which indicates the intensity of the certain symptom that you experienced over the PAST WEEK. There are no right or wrong answers – there are only the right ones, that is your feelings. Do not spend too much time on any of the statements.

Q: I found it hard to wind down: * *(type: choice)*
A: one of the following:
Never
Sometimes
Often
Always/Almost always

Q: I was aware of dryness of my mouth* *(type: choice)*
A: one of the following:
Never
Sometimes
Often
Always/Almost always

Q: I could not seem to experience any positive feeling at all* *(type: choice)*
A: one of the following:
Never
Sometimes
Often
Always/Almost always

Q: I experienced breathing difficulty (e.g., excessively rapid breathing, breathlessness in the absence of physical exertion)* *(type: choice)*
A: one of the following:
Never
Sometimes
Often
Always/Almost always

Q: I found it difficult to work up the initiative to do things* *(type: choice)*
A: one of the following:
Never
Sometimes
Often
Always/Almost always

Q: I tended to over-react to situations* *(type: choice)*
A: one of the following:
Never
Sometimes
Often
Always/Almost always

Q: I experienced trembling (e.g., in the hands)* *(type: choice)*
A: one of the following:
Never
Sometimes
Often
Always/Almost always

Q: I felt that I was using a lot of nervous energy* *(type: choice)*
A: one of the following:
Never
Sometimes
Often
Always/Almost always

Q: I was worried about situations in which I might panic and make a fool of myself* *(type: choice)*
A: one of the following:
Never
Sometimes
Often
Always/Almost always

Q: I felt that I had nothing to look forward to* *(type: choice)*
A: one of the following:
Never
Sometimes
Often
Always/Almost always

Q: I found myself getting agitated* *(type: choice)*
A: one of the following:
Never
Sometimes
Often
Always/Almost always

Q: I found it difficult to relax* *(type: choice)*
A: one of the following:
Never
Sometimes
Often
Always/Almost always

Q: I felt down-hearted and blue * *(type: choice)*
A: one of the following:
Never
Sometimes

Often
Always/Almost always

Q: I was intolerant of anything that kept me from getting on with what I was doing* *(type: choice)*
A: one of the following:
Never
Sometimes
Often
Always/Almost always

Q: I felt I was close to panic* *(type: choice)*
A: one of the following:
Never
Sometimes
Often
Always/Almost always

Q: I was unable to become enthusiastic about anything* *(type: choice)*
A: one of the following:
Never
Sometimes
Often
Always/Almost always

Q: I felt I was not worth much as a person* *(type: choice)*
A: one of the following:
Never
Sometimes
Often
Always/Almost always

Q: I felt that I was rather touchy * *(type: choice)*
A: one of the following:
Never
Sometimes
Often
Always/Almost always

Q: I was aware of the action of my heart in the absence of physical exertion (e.g., sense of heart rate increase, heart missing a beat) * *(type: choice)*
A: one of the following:
Never
Sometimes
Often
Always/Almost always

Q: I felt scared without any good reason* *(type: choice)*
A: one of the following:
    Never
    Sometimes
    Often
    Always/Almost always

Q: I felt that life was meaningless* *(type: choice)*
A: one of the following:
    Never
    Sometimes
    Often
    Always/Almost always
